# “Tear Drops in the Duodenum": Uncommon Cause of Iron Deficiency Anemia in Adults

**DOI:** 10.7759/cureus.5532

**Published:** 2019-08-30

**Authors:** Isma N Javed, Rutaba Tajammal, Sardar H Ijaz, Nazir Ahmad, Sultan Mahmood

**Affiliations:** 1 Internal Medicine, University of Oklahoma Health Sciences Center, Oklahoma City, USA; 2 Internal Medicine, Saint Anthony Hospital, Oklahoma City, USA; 3 Gastroenterology, Marshfield Clinic, Marshfield, USA

**Keywords:** iron deficiency anemia, anemia, giardiasis, fatigue, geriatric patients, giardia lamblia, metronidazole, iron deficiency

## Abstract

A 64-year-old man presented to the internal medicine resident clinic with fatigue and abdominal pain of six-month duration. He did not have diarrhea, hematemesis, melena, or hematochezia. Physical examination was unremarkable. Laboratory findings were consistent with iron deficiency anemia. Upper and lower gastrointestinal (GI) endoscopies revealed normal findings. Duodenal biopsy showed trophozoites (tear-drop-shaped) morphologically consistent with Giardia duodenalis. He was prescribed metronidazole and iron replacement therapy, with a resultant improvement in symptoms as well as lab values at the four-month follow-up visit.

## Introduction

Giardia duodenalis (also known as Giardia lamblia) is a protozoan parasite that commonly affects young children, travelers [1], and immunocompromised individuals. Giardiasis is an important cause of food-borne/water-borne diseases [2]. The fecal-oral route is the most common mode of transmission [3]. It is more common in children than in adults [4]. Giardia has been reported as a leading parasite causing enteric infections in the United States [5]. We report a case of giardiasis in an adult man, without typical symptoms or travel history, manifesting as iron deficiency anemia.

## Case presentation

**Figure 1:**
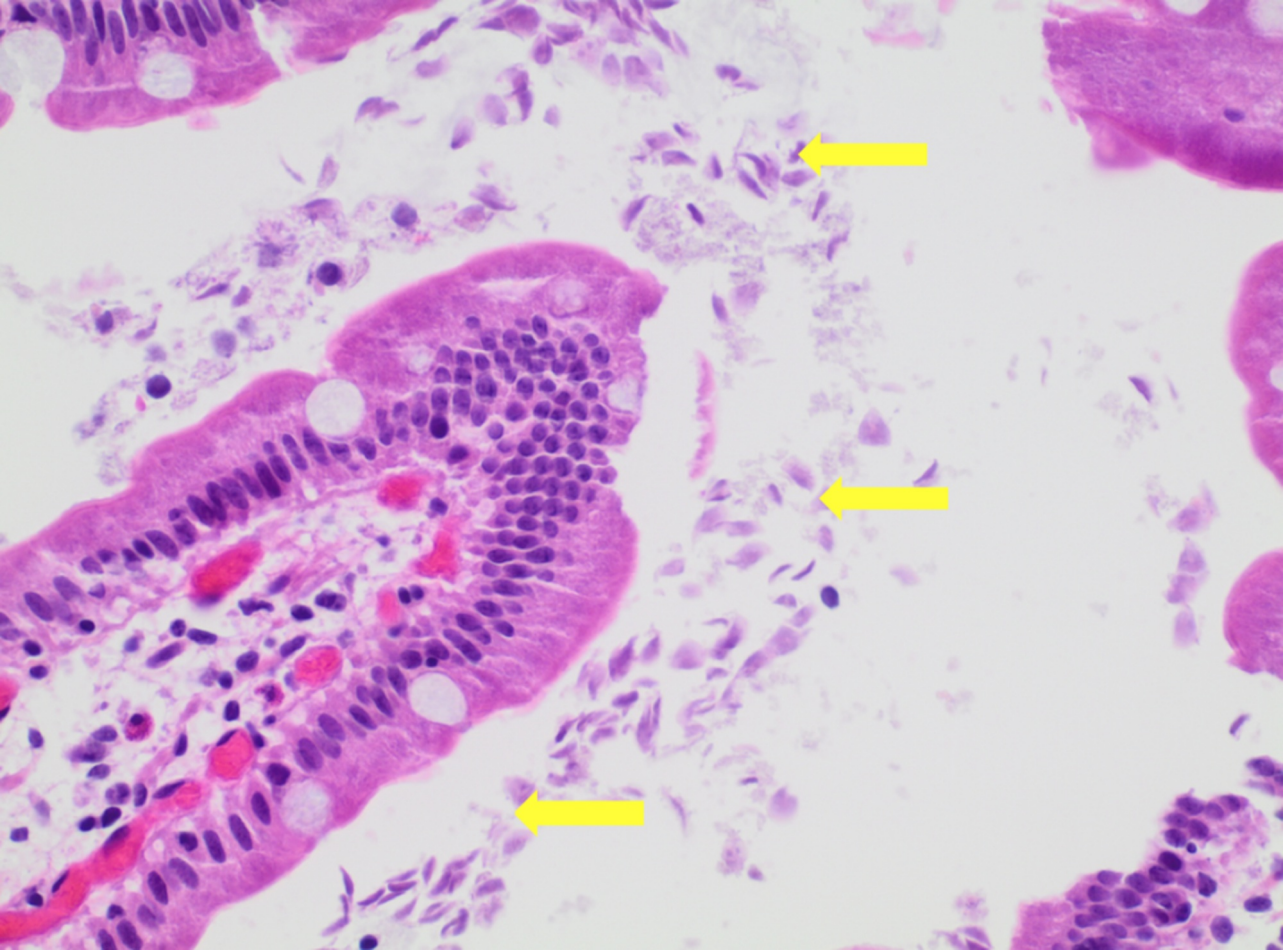
H & E at 40x showing Giardia duodenalis surrounding the entire villus

**Figure 2:**
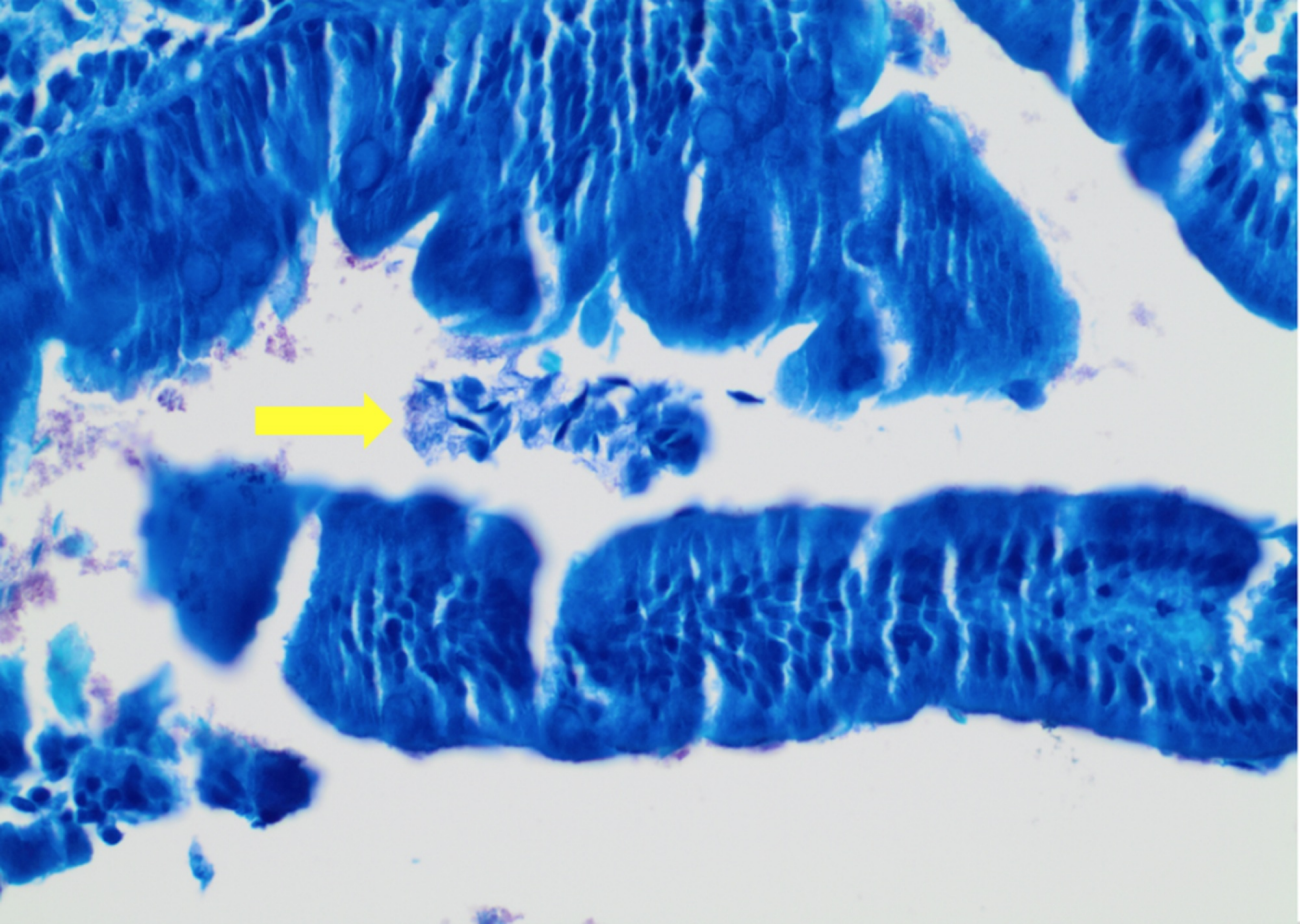
Giemsa stain at 40x showing tear-drop-like Giardia

**Figure 3:**
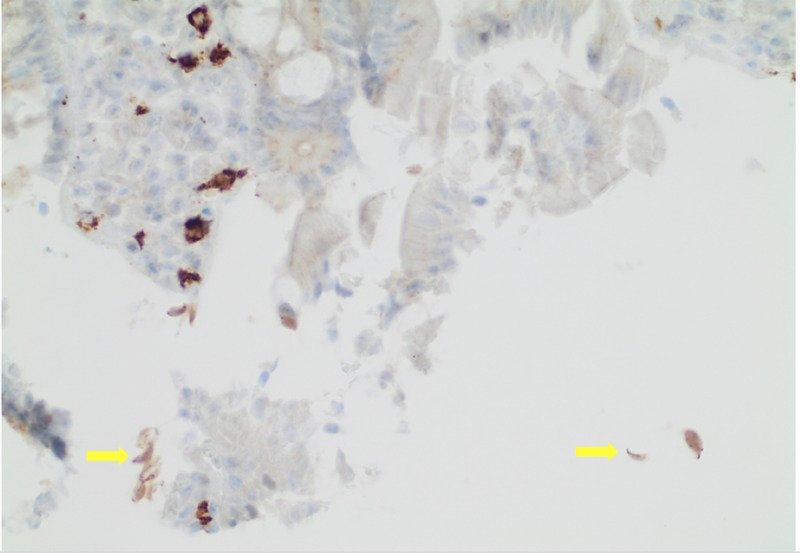
CD 117 at 40x. Arrows showing fall-leaves-like Giardia

A 64-year-old man with a medical history significant for hypertension and tobacco abuse presented to the internal medicine clinic, complaining of fatigue and abdominal pain of six-month duration. He denied any hematemesis, melena, or hematochezia. The physical exam was unremarkable. Laboratory findings were significant for microcytic hypochromic anemia with hemoglobin level 8 g/dl, mean cell volume 70.6 fl, and mean cell hemoglobin 21.1 pg. Serum vitamin B12 and folate were normal. The iron profile panel showed a low iron level, low iron saturation, and low ferritin, with an increased total iron-binding capacity. The celiac panel was negative. He was referred for an esophagogastroduodenoscopy (EGD) and a colonoscopy. The colonoscopy revealed normal findings. EGD showed normal mucosa. The biopsies from the stomach were negative for Helicobacter pylori (H. pylori). Duodenal biopsy showed normal mucosa and trophozoite forms (tear-drop-shaped) morphologically consistent with Giardia duodenalis (Figures 1-2). CD117 and Giemsa stains also showed positivity for Giardia duodenalis (Figure 3). The patient was treated with a one-week course of 400 mg metronidazole three times a day. He was also given a prescription for 325 mg ferrous sulfate once daily. At the four-month follow-up, the patient reported a marked improvement in his symptoms. Repeat complete blood count showed hemoglobin of 14 g/dl.

## Discussion

Giardiasis, an enteric infection caused by the protozoan parasite Giardia, was first described by Leeuwenhoek in 1681, who observed the parasite in his own stool [6]. Giardia duodenalis is the third leading cause of diarrheal illness worldwide mostly affecting the pediatric population. It is an important cause of daycare center outbreaks. In developing countries, prevalence has been reported as high as 20-40 percent. High-risk groups include infants, young children, immunocompromised patients, international travelers, and patients with cystic fibrosis. On the contrary, Giardia infection is not frequently encountered in developed countries. Compared to adults, it is more frequently reported in children aged one to nine years [7]. The reported prevalence of giardiasis in adults was reported as 0.11% in a cross-sectional study [8]. Male sex, southern region, and the presence of H. pylori have been independently associated with giardiasis [8]. Giardia has two morphological forms. Cysts are the infectious form excreted in the stool. Following cyst ingestion, the trophozoite form is released into the small intestine with some traveling to the large intestine where these are converted to infectious cyst form. Three routes of transmission have been described: water-borne, food-borne, and fecal-oral routes. The most commonly reported symptoms include diarrhea, abdominal pain, nausea, vomiting, anorexia, and weight loss [9]. Asymptomatic infection also occurs, whereas chronic carriers can present with delayed growth and development, weight loss, and fatigue owing to the malabsorption of vitamins and other dietary nutrients. The diagnostic tests include antigen detection assays, nucleic acid detection assays, and stool examination [10]. Out of these tests, stool microscopy is the least sensitive. Treatment is mainly aimed toward supportive care and antimicrobial therapy. Nitroimidazole drugs, i.e., metronidazole and tinidazole, are a highly effective class of drugs. A five- to seven-day course of metronidazole or a single dose of tinidazole can cure more than 90% of patients [11].

## Conclusions

The clinical presentation of giardiasis varies but it should be kept in the differential diagnoses in appropriate clinical settings such as in adult patients with unexplained iron deficiency anemia. This case emphasizes the fact that giardiasis can occur in the adult population without typical exposure (travel history) and presentation. Although not routinely recommended, EGD with duodenal biopsy can be considered in cases with a high degree of suspicion for giardiasis. Treatment is usually very effective, as mentioned earlier. Therefore, prompt diagnosis is critical.
